# Restoration of correct β^IVS2-654^-globin mRNA splicing and HbA production by engineered U7 snRNA in β-thalassaemia/HbE erythroid cells

**DOI:** 10.1038/s41598-019-43964-3

**Published:** 2019-05-21

**Authors:** Tiwaporn Nualkaew, Natee Jearawiriyapaisarn, Suradej Hongeng, Suthat Fucharoen, Ryszard Kole, Saovaros Svasti

**Affiliations:** 1 Thalassemia Research Center, Institute of Molecular Biosciences, Mahidol University, Nakhon Pathom, Thailand; 2 Departments of Pediatrics, Faculty of Medicine Ramathibodi Hospital, Mahidol University, Bangkok, Thailand; 3 Ercole Consulting, Chapel Hill, NC 27514 USA; 4 Department of Biochemistry, Faculty of Science, Mahidol University, Bangkok, Thailand

**Keywords:** RNAi, Molecular medicine

## Abstract

A cytosine to thymine mutation at nucleotide 654 of human β-globin intron 2 (β^IVS2-654^) is one of the most common mutations causing β-thalassaemia in Chinese and Southeast Asians. This mutation results in aberrant β-globin pre-mRNA splicing and prevents synthesis of β-globin protein. Splicing correction using synthetic splice-switching oligonucleotides (SSOs) has been shown to restore expression of the β-globin protein, but to maintain therapeutically relevant levels of β-globin it would require lifelong administration. Here, we demonstrate long-term splicing correction using U7 snRNA lentiviral vectors engineered to target several pre-mRNA splicing elements on the β^IVS2-654^-globin pre-mRNA such as cryptic 3′ splice site, aberrant 5′ splice site, cryptic branch point and an exonic splicing enhancer. A double-target engineered U7 snRNAs targeted to the cryptic branch point and an exonic splicing enhancer, U7.BP + 623, was the most effective in a model cell line, HeLa IVS2-654. Moreover, the therapeutic potential of the vector was demonstrated in erythroid progenitor cells derived from β^IVS2-654^-thalassaemia/HbE patients, which showed restoration of correctly spliced β-globin mRNA and led to haemoglobin A synthesis, and consequently improved thalassaemic erythroid cell pathology. These results demonstrate proof of concept of using the engineered U7 snRNA lentiviral vector for treatment of β-thalassaemia.

## Introduction

β-Thalassaemia is a common genetic disorder of haemoglobin synthesis, caused by defects in β-globin chain production. This results in deficiency of β-globin chains and adult haemoglobin (HbA), leading to chronic anaemia and a shortened life expectancy^1^. β-Thalassaemia is caused by more than two hundred mutations in the β-globin gene yet less than fifteen cause the majority of β-thalassaemia cases worldwide. Mutation induced aberrant splicing, such as mutations in the intron 2 of the β-globin gene at nucleotides 654, 705, or 745 (β^IVS2-654^, β^IVS2-705^ and β^IVS2-745^-globin), is one of the important molecular mechanisms of the defect in human β-globin gene expression. The mutation β^IVS2-654(C>T)^-globin (HBB:c316-197C > T) is one of the most common β-thalassaemic mutations in Chinese and Southeast Asians that affects β-globin pre-mRNA splicing. A cytosine to thymine substitution at nucleotide 654 of β-globin intron 2 creates an aberrant 5′ splice site at nucleotide 652 and activates a cryptic 3′ splice site at nucleotide 579. The resulting mRNA is incorrectly spliced and retains a 73 nucleotide fragment of intron 2. A frameshift in the coding sequence in the aberrant mRNAs prevents their translation into full-length β-globin, leading to β-thalassaemia^2^.

Recently, two splice-switching oligonucleotides (SSO), eteplirsen and nusinersen for Duchenne muscular dystrophy and spinal muscular atrophy, repectively were approved by the U.S. Food and Drug Administration^3,4^ as sequence specific modulators of gene expression. SSOs block splicing elements in the pre-mRNA, forcing the splicing machinery to re-select the correct splice sites and thereby correct pre-mRNA splicing. The ability of SSO to block aberrant splicing and restore correct splicing has been shown in several genetic diseases.

Correction of pre-mRNA splicing with SSO has been shown in *in vitro* study of erythroid progenitors obtained from β-thalassaemia patients^5,6^ and β-thalassaemic mice, carrying the human β^IVS2-654^-thalassaemia gene^7^. In addition, administration of SSO to β^IVS2-654^-thalassaemic mice led to restoration of correct β-globin pre-mRNA splicing, increasing haemoglobin levels and improving haematological parameters^7^. However, the SSO treatment of patients would require life-long administration to maintain therapeutically relevant levels of β globin. To address this issue and to generate long-term effects, the SSO sequences were incorporated into the murine U7 small nuclear RNA (snRNA) as a vehicle to maintain stable levels of the SSO and consequently modulate pre-mRNA splicing in target cells. This approach resulted in restoration of β-globin pre-mRNA correct splicing for at least 6 months in HeLa cells expressing β^IVS2-654^-, β^IVS2-705^- and β^IVS2-745^-globin (HeLa IVS2-654, HeLa IVS2-705 and HeLa IVS2-745, respectively)^8–10^. Interestingly, the increase in correct splicing was only about 3% of total in HeLa IVS2-654 cells, in comparison to nearly 100% in HeLa IVS2-745 cells^10^, indicating that the effects are dependent on the response of the target sequence.

In this study, we generated a series of engineered U7 snRNAs targeted to several elements involved in the aberrant splicing of β^IVS2-654^-globin pre-mRNA and evaluated their long-term effects on β^IVS2-654^-globin pre-mRNA splicing in relevant cell line models. We also show that lentiviral vectors carrying the optimal SSO sequences restored correct β^IVS2-654^-globin pre-mRNA splicing and HbA production in erythroid progenitor cells from β^IVS2-654^-thalassaemia/HbE patients which led to phenotypic improvements of β-thalassaemic erythroblast.

## Results

### Effective correction of β^IVS2-654^-globin pre-mRNA splicing by engineered U7 snRNAs

Among the three thalassaemia mutations in intron 2 of the β-globin gene, IVS2-654, −705, and −745, the β^IVS2-654^ mutation is most resistant to correction by SSO approach^11^. Moreover, in previous work, the lentiviral vector carrying an engineered U7 snRNA targeted to an exonic splicing enhancer (ESE) at nucleotide position 623 of β-globin intron 2 (U7.623) could barely restore correct splicing in HeLa IVS2-654 cells^10^. Here we set out to achieve much higher efficiency. A series of engineered U7 snRNAs were generated and evaluated in two model cell lines, HeLa IVS2-654 and HeLa EGFP-654. The HeLa IVS2-654 cells harbour the β^IVS2-654^-thalassaemic mutation (Fig. S1A). While, the HeLa EGFP-654 cells carry EGFP gene in which the coding sequence is interrupted by intron 2 of the β^IVS2-654^ thalassaemic gene, consequently aberrantly splice of EGFP pre-mRNA (Fig. S1B).

**Figure 1:**
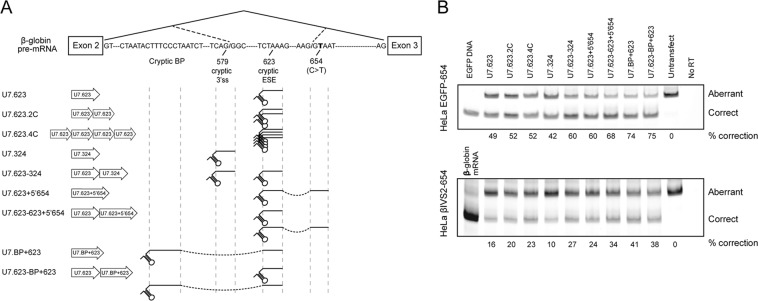
Identification of target site for correction of β^IVS2-654^-globin pre-mRNA splicing. (**A**) Schematic diagram of target sites for the engineered U7 snRNAs on β^IVS2-654^-globin pre-mRNA. (**B**) Correction of aberrant splicing by engineered U7 snRNAs in HeLa EGFP-654 and HeLa IVS2-654 cells. Total RNA of cells transiently transfected with engineered U7 snRNA was analysed by semi-quantitative RT-PCR labelled with Cy5-dCTP. The percentage of correctly spliced mRNA is indicated below the lanes.

First, we attempted to increase expression levels of U7.623 snRNA by increasing the copy number of U7.623 to two or four copies of U7.623 in a head to tail orientation (U7.623.2C and U7.623.4C) (Fig. 1A). This approach increased correct splicing of EGFP mRNA and β-globin mRNA only slightly as compared to a single copy of U7.623 in HeLa EGFP-654 and HeLa IVS2-654 (Fig. 1B). To improve on this result a plasmid carrying two different U7 snRNAs, U7.623 and U7.324, which target the cryptic 3′ splice sites, (U7.623-324) was tested. This construct led to increased correction over either U7.623 or U7.324 alone (Fig. 1B). This suggested that two different SSO, binding to two different sequences involved in aberrant splicing of the same pre-mRNA, enhance the efficiency of splicing correction. This was confirmed with a U7 snRNA that targets the ESE and the aberrant 5′ splice site at nucleotide 654 (U7.623 + 5′654) or ESE and a cryptic branch point (U7.BP + 623) which significantly improved correction efficiency in both HeLa EGFP-654 and HeLa IVS2-654 (Fig. 1B). Further combinations, of two U7 snRNAs, U7.623 and double-target U7 snRNA U7.623 + 5′654 (U7.623-623 + 5′654) or U7.BP + 623 (U7.623-BP + 623) increased correction efficiency only slightly over the double-target U7 snRNA alone (Fig. 1B).

### Stable correction of aberrant splicing in HeLa EGFP-654 by U7.BP + 623 snRNA lentiviral vector

**Figure 2:**
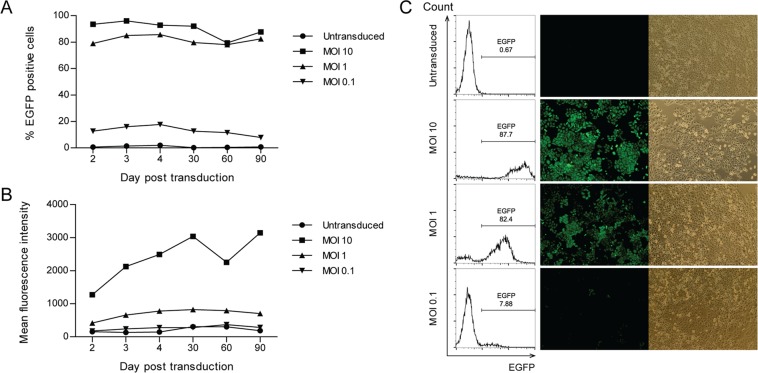
Stable correction of aberrant splicing in HeLa EGFP-654 cells by U7.BP + 623 snRNA lentiviral vector. (**A**) Percentage of EGFP + cells and (**B**) mean fluorescence intensity of EGFP of HeLa EGFP-654 cells following U7.BP + 623 snRNA lentiviral vector transduction for 90 days. (**C**) Flow cytometry plot and fluorescence microscopy of identically transduced-HeLa EGFP-654 cells at 60 days post transduction. Fluorescence (middle panel) and phase contrast (right panel) images are shown.

The most effective of the engineered U7 snRNAs, U7.BP + 623, was investigated to determine if it could achieve long-term splicing correction. Transduction of HeLa EGFP-654 cells with U7.BP + 623 snRNA lentiviral vector at MOI 0.1–10 showed a dose-dependent restoration of EGFP expression (Fig. 2A,B). At MOI of 10, over 90% of the cells were transduced and expressed the highest level of EGFP. The EGFP expression was not significantly changed during continuous culture for 3 months. As confirmation of the flow cytometry analysis, fluorescence microscopy analysis of EGFP was performed at 2 months post-transduction (Fig. 2C). The experiments demonstrated long-term correction by the U7.BP + 623 lentiviral vector in HeLa EGFP-654 cells. In addition, an unchanged fluorescence intensity and percentage of EGFP-expressing cells implied that no cytotoxic effects resulted from the lentiviral transduction or from U7.BP + 623 snRNA expression in target cells.

### Restoration of correct β-globin mRNA splicing and HbA production by U7.BP + 623 snRNA lentiviral vector in thalassaemia patient erythroid progenitor cells

**Figure 3:**
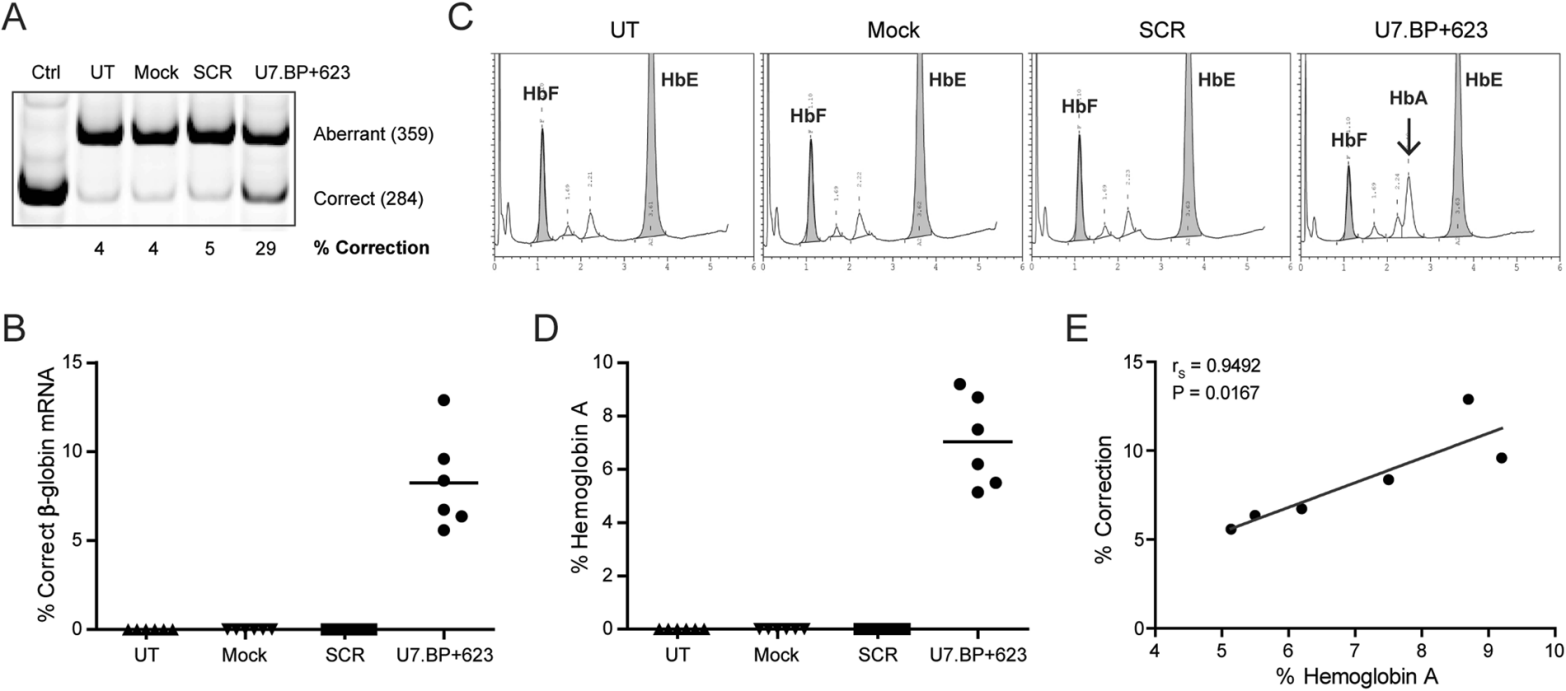
Restoration of correct β-globin mRNA splicing and HbA production by U7.BP + 623 snRNA lentiviral vector in erythroid cells. Correction of β-globin mRNA splicing and HbA production in β^IVS2-654^-thalassaemia/HbE erythroid progenitor cells transduced with U7.BP + 623 snRNA lentiviral vector. (**A**,**B**) correction of β-globin mRNA splicing was analysed by semi-quantitative RT-PCR labelled with Cy5-dCTP. (**C**,**D**) HbA production was analysed by HPLC. Haemoglobin of normal subject was used as control (normal); (**E**) Correlation analysis of correctly spliced β-globin mRNA and HbA. Correlation analysis was performed using Spearman Rank method (p < 0.05). UT, untransduced cells; Mock, mock control; SCR, scramble control; U7.BP + 623, U7.BP + 623 snRNA lentiviral vector; HbF, fetal haemoglobin; HbA, adult haemoglobin; HbE, haemoglobin E. Arrows indicate restored HbA.

To evaluate the therapeutic potential of the U7.BP + 623 snRNA lentiviral vector in the ultimate target cell, CD34 + haematopoietic progenitor cells derived from β^IVS2-654^-thalassaemia/HbE patient were transduced with U7.BP + 623 snRNA or the scrambled sequence of U7.BP + 623 (SCR) in a lentiviral vector. The lentiviral vector backbone served as a mock control (See materials and methods for details). Average vector copy number (VCN) of U7.BP + 623 snRNA lentiviral vector transduced cells, examined by qPCR using specific primer to U7.BP + 623 snRNA, was 0.28 copies per cell (ranged from 0.19 to 0.37). Correction of aberrant splicing was determined by semi-quantitative PCR analysis on day 10 of erythroid cell culture, which predominantly contains the basophilic normoblast and early polychromatophilic normoblast stages of differentiating erythroid cells. The level of correctly spliced β-globin mRNA of U7.BP + 623 snRNA lentiviral vector transduced cells was 8.25 ± 2.7% (Fig. 3A,B). Importantly, analysis of haemoglobin composition on day 14 of erythroid culture showed 7.04% ± 1.69% restoration of HbA production in the U7.BP + 623 snRNA lentiviral vector transduced cells (Fig. 3C,D). The percentage of HbA was significantly correlated with the percentage of correctly spliced β-globin mRNA (r_s_ = 0.9495, *p* = 0.0167) (Fig. 3E). As expected, there was no correctly spliced β-globin mRNA in control cells. Clearly, the engineered U7.BP + 623 snRNA lentiviral vector not only promoted restoration of correct splicing of β^IVS2-654^-globin mRNA, but also restored HbA production in erythroid progenitor cell derived from β^IVS2-654^-thalassaemia/HbE patients.

**Figure 4:**
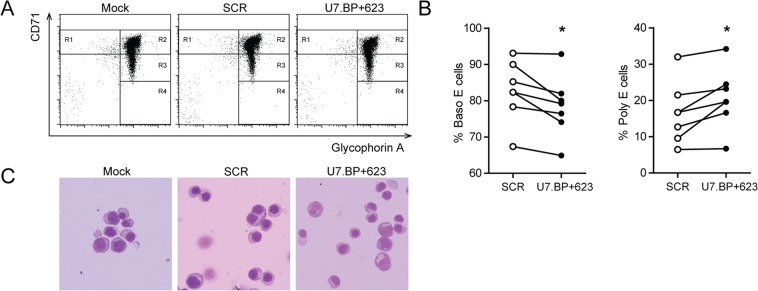
Pathological improvement of thalassaemic erythroid cells following U7.BP + 623 snRNA lentiviral vector treatment. (**A**,**B**) Flow cytometry analysis of erythroid differentiation using antibodies against human transferrin receptor (CD71) and glycophorin A (CD235a). R1 represents proerythroblast, R2 represents basophilic erythroblast (Baso **E**), R3 represents polychromatophilic erythroblast (Poly **E**), and R4 represents orthochromatophilic erythroblast (Ortho **E**). Statistical analysis was performed using a paired t-test (Wilcoxon signed rank test) (p < 0.05). (**C**) Cell morphology was assessed by modified Wright’s-Giemsa staining. Mock, mock control; SCR, scramble control; U7.BP + 623, U7.BP + 623 snRNA lentiviral vector.

To determine whether increased production of correctly-spliced β-globin mRNAs and HbA are therapeutically relevant, erythroid differentiation was assessed on day 12 of culture by flow cytometry analysis using CD235a and CD71 surface markers (Fig. 4A). A significantly increased number of polychromatophilic and orthochromatic erythroblasts and a decreased number of basophilic erythroblasts was observed in cells treated with U7.BP + 623 snRNA lentiviral vector as compared with the SCR vector (Fig. 4B). The improvement of erythroid differentiation was also clearly evident by light microscopy (Fig. 4C). These results indicate that the maturation of β-thalassaemia/HbE erythroid progenitor cells was improved after treatment with the U7.BP + 623 snRNA lentiviral vector.

## Discussion

Successful repair of β-globin pre-mRNA splicing defect by synthetic SSO in erythroid cells from β-thalassaemia/HbE patients was demonstrated^5,6,12^. However, its clinical application is limited by short-term effectiveness and the requirement for lifelong periodic administration of the SSO, especially for a chronic disease like thalassaemia. Here, we engineered lentiviral vectors that stably express U7 snRNA carrying the SSO that restores the correct splicing of β^IVS2-654^-globin pre-mRNA and achieved a long-term effect.

The major obstacle of SSO therapy in clinical application is delivery and stability of the SSO in target tissues. Several viral vector mediated SSO therapies have been developed. Viral vectors such as adenovirus vectors, adeno-associated virus (AAV) vectors and lentivirus vectors carrying SSOs have been investigated in disease model cell lines, primary cells from patient or animal models. AAV vectors carrying an engineered U7 snRNAs were used to mediate exon skipping in mouse models of several diseases including spinal muscular atrophy, Duchenne muscular dystrophy, hypertrophic cardiomyopathy and familial amyotrophic lateral sclerosis^13–16^. However, the non-integrative characteristic of this vector results in the gradual loss of the vector from dividing cells. In order to correct splicing defects in β-thalassaemia, we aimed to target haematopoietic stem cells, which divide rapidly and thus a non-integrative AAV vector is not suitable. Therefore, an integrative lentiviral vector seems to be a choice as a vector for long-term correction by snRNA-mediated SSO in erythropoietic cells. The current success of β-thalassaemia gene therapy using a lentiviral vector^17^ renders the possibility of clinical application of the lentiviral vector-snRNA-mediated splicing correction approach.

Embedding the SSO in an U7 snRNP has several advantages over the application of naked RNAs. The U7 snRNP are expressed at high levels and are located in the nucleus where RNA splicing takes place. Furthermore, 5′ capping, secondary structure and binding with small nuclear ribonucleoprotein (snRNP)–specific proteins render them resistant to nuclease degradation and promises continuous expression. These advantages make SSO-embedded U7 snRNAs attractive, as showed by the successful modulation of pre-mRNA splicing in Duchenne muscular dystrophy^16,18^, spinal muscular atrophy^15^, and β-thalassaemia^10,19^.

In this work, we chose a challenging target, β^IVS2-654^-globin mutation and found that a double-target constructs were the most effective, presumably by acting synergistically to block the aberrant splicing pathway. This is likely due to a looping or cross-linked structure of the target pre-mRNAs that are bound by the double-target constructs which may strongly inhibit the aberrant splicing pathway^9^. Masking at the cryptic branch point and the ESEs in the aberrant exon by U7.BP + 623 snRNA prevented or disrupted formation of the spliceosome complex at these sites and therefore the aberrant splicing was effectively inhibited. Since the U7.BP + 623 snRNA targeted the cryptic branch point and ESEs utilised by the splicing machinery in the aberrant splicing of β^IVS2-654^, β^IVS2-705^ and β^IVS2-745^-globin pre-mRNAs, this construct would be useful for correction of splicing for all three mutations expanding the number of benefiting patients.

In this study, long-term correction by a lentiviral vector-snRNA-mediated SSO approach was shown in a disease model cell line carrying the IVS2-654 mutation. Importantly, restoration of correct β-globin mRNA splicing and HbA synthesis by U7.BP + 623 snRNA lentiviral vector was demonstrated in erythropoietic cells derived from β-thalassaemia patients and improvement of erythroid cell differentiation was observed. The potential of this approach was previously demonstrated using a U7 snRNA-based vector targeted to the β^E^-globin pre-mRNA, which restores correct splicing of β^E^-globin pre-mRNA and improves the pathology of patients with β^E^-thalassaemia^19^. Even though complete splicing correction was not achieved in this work, the approximately 10% of correction resulted in phenotypic improvement and should improve the balance of α/β-globin. Notably this result twice as good as previously observed 5% levels of chimeric haemoglobin achieved by IV injection of SSOs in β^IVS2-654^ thalassaemic mice^7^. The low VCN (0.19 to 0.37 copy per cell) may lessen the effect on restoring of β-globin correct splicing, thus higher VCN is required for higher level of the engineered U7 snRNA expression to achieve the therapeutic level of HbA in β-thalassaemia. In addition, erythroid specific control, may also resulted in higher levels of splicing correction.

Here, we provided a proof of concept using the engineered U7 snRNA to mediate correct splicing of β^IVS2-654^-globin pre-mRNA as an alternative option for treatment of β-thalassaemia. The engineered U7 snRNA was under control of RNA polymerase II promoter which would resulted in expression in all haematopoietic lineages if delivered to haematopoietic stem cells. Thus, for clinical translation, high-level and erythroid-specific expression of the engineered U7 snRNA will be necessary. The β-globin gene promoter and its mini-locus control region (LCR) would be the compelling regulatory elements for erythroid-specific expression of the engineered U7 snRNA as their successfully used in several gene therapy vectors for β-thalassaemia and sickle cell disease clinical trials such as BB305 vector^17,20,21^, TNS9.3.55 vector (NCT01639690, Clinicaltrials.gov), and GLOBE vector^22^. The minimal β-globin proximal promoter linked to β-globin LCR (HS2-HS3), which is regulatory element of the GLOBE vector, have been successfully used for control expression of shRNA targeting BCL11A for upregulation of fetal haemoglobin (HbF) in preclinical study^23^ and are being investigated in clinical trials (NCT03282656, Clinicaltrials.gov). Therefore, erythroid-specific expression system of SSO would render a lentiviral vector-snRNA-mediated SSO approach as a promising alternative approach for β-thalassaemia gene therapy and it might prevent negative effects of the SSO in non-target cells.

The first successful gene therapy trial for β-thalassemia was able to achieve transfusion independence in one adult patient with β°-thalassaemia/HbE^24^. Two phase 1–2 studies in 22 patients, using the BB305 vector (NCT01745120 and NCT02151526, Clinicaltrials.gov) showed that most of patients with non-β^0^/β^0^-thalassaemia genotype and three of nine β^0^/β^0^-thalassaemia patients became transfusion independent after infusion, while six of β^0^/β^0^-thalassaemia patients continue receiving blood transfusion but at decreased frequency. VCN in peripheral blood mononuclear cells ranged from 0.1 to 4.2 copies per cell^17^. Recently, gene therapy trials using GLOBE vector (NCT02453477, Clinicaltrials.gov) showed three of four paediatric patients achieved transfusion independence and three adult patients had reduced transfusion requirement with a VCN in erythroid precursors ranged from 0.10 to 1.97 copies per cell^22^. While clinical trials using TNS9.3.55 vector (NCT01639690, Clinicaltrials.gov) is still under investigated. These showed promising results for β-thalassaemia gene therapy by β-globin gene transfer method. However, the severe β^0^/β^0^-thalassaemia patients were less success by the treatment. A phase 3 study of the BB305vector (NCT02906202) has been initiated to increase VCN and proportion of transduced cells^21^. Increasing of VCN not only directly increased level of the therapeutic haemoglobin but also increased risk of genotoxicity causing from random integration of vector, although no adverse effect was reported yet. Alternatively, erythroid-specific knockdown of BCL11A using shRNA to reactivating HbF production of sickle cells disease could be a therapeutic option that would be beneficial in β-thalassaemia patients. The outcome of this approach remains to be seen. In addition, the strategy of the engineered U7 snRNA that reported here may be a new alternative approach for β-thalassaemia gene therapy, even though improvements of the vector system are still required.

While bone marrow transplantation is curative for thalassaemia in some instances, it is limited by the scarcity of suitably matched donors and facilities, and the high cost of the procedure. Moreover, even when available, bone marrow transplantations can carry significant risks. The advantage of the SSO approach is that the correction by SSO occurs in the β-globin pre-mRNA transcribed from the native β-globin locus thus precluding overexpression of β-globin mRNA. Additionally, engineered U7 snRNA mediated splicing correction can be implemented in numerous other diseases caused by aberrant pre-mRNA splicing.

## Materials and Methods

### Recombinant plasmid constructs

The U7SmOPT plasmid carries a modified murine U7 snRNA gene in which the consensus Sm sequence (SmOPT) replace the U7-specific Sm binding site under the control of its natural promoter and terminator^25^. The histone pre-mRNA 3′ processing site complementary nucleotide sequence of the U7 snRNA was replaced with a series of SSO sequences complementary to the β^IVS2-654^-globin pre-mRNA (Fig. S2) by the PCR-based mutagenesis method using a QuikChange Site-Directed Mutagenesis Kit (Stratagene, La Jolla, CA).

The engineered U7 snRNA gene was cloned into the pLL3.7 lentiviral vector which carries the enhanced green fluorescence protein (EGFP) reporter gene^26^ or pLL-Puro in which the EGFP gene was replaced by a puromycin resistance gene^19^ to generate lentiviral vectors expressing the engineered U7 snRNAs.

### Cell lines and transient transfections

HeLa IVS2-654 cells carry the β^IVS2-654^ thalassaemic mutation under the control of the cytomegalovirus promoter (CMV)^27^. HeLa EGFP-654 cells carry the coding sequence of EGFP in which the intron 2 of the β^IVS2-654^ thalassaemic gene is inserted at nucleotide 105^28^. HeLa cells were maintained in minimum essential medium modified for suspension cultures (S-MEM; Gibco, Thermo Fisher Scientific, Waltham, MA) supplemented with 5% fetal bovine serum (FBS; Gibco), 5% horse serum (Gibco), 2 mM L-glutamine (Gibco), 50 μg/ml gentamicin (Sigma-Aldrich, Merck, Darmstadt, Germany), 200 μg/ml kanamycin (Sigma-Aldrich) at 37 °C 5% CO_2_. HeLa cells were transfected with the engineered U7 snRNA plasmids using Lipofectamine 2000 (Invitrogen; Thermo Fisher Scientific).

### Erythroid progenitor cell culture

This study was performed in accordance with the Helsinki declaration and was approved by the Mahidol University Institutional Review Board (MU-IRB), approval number 2013/022.1103. Written informed consent was obtained from all individual participants included in the study. Human CD34 + cells were isolated from peripheral blood obtained from six β^IVS2-654^-thalassaemia/HbE patients. Briefly, peripheral blood mononuclear cells were isolated by centrifugation on Lymphoprep (Axis-Shield, Oslo, Norway) and subsequently enriched for CD34 + cells by immunomagnetic separation using the CD34 MicroBead kit (Miltenyi Biotec, Bergisch Gladbach, Germany) as per the manufacturer’s instructions. The CD34 + cells were cultured for 14 days in two-phase liquid culture. The first phase, cells were cultured for 4 days in Iscove’s Modified Dulbecco Medium (IMDM; Gibco) supplemented with 20% FBS (Sigma-Aldrich), 100 U/mL penicillin/streptomycin (Gibco), 300 μg/mL holo-human transferrin (PromoCell, Heidelberg, Germany), 50 ng/mL human stem cell factor (Miltenyi Biotec), 10 ng/mL human interleukin-3 (Miltenyi Biotec), 2 U/mL erythropoietin (Epo; EPREX; Janssen-Cilag, Schaffhausen, Switzerland). The second phase, cells were subsequently stimulate towards erythrocytes differentiation for 10 days in IMDM containing 20% FBS, 100 U/mL penicillin/streptomycin, 300 μg/mL holo-human transferrin and 5 U/mL Epo.

### Lentiviral vector production and transduction

The lentiviral vector was produced by transient co-transfection of HEK293T cells with a four-plasmid packaging system and concentrated using Lenti-X Concentrator (Clontech Laboratories, Mountain View, CA) as previously described^29^.

For transduction of HeLa EGFP-654 cells, the cells were transduced with the lentiviral vectors at various multiplicity of infection (MOI) in the presence of 4 μg/mL polybrene (Hexadimethrine bromide; Sigma-Aldrich).

For transduction of CD34 + cells, the cells were transduced with lentivirus at an MOI of 150 in the presence of 8 μg/mL polybrene at day 3 of first phase. Transduced cells were selected by treated with 0.5 μg/mL puromycin (Clontech Laboratories) at 72 hr after transduction and continued for 2 days.

### Analysis of EGFP expression

EGFP expression of lentiviral transduced HeLa EGFP-654 cells was determined using FACSCalibur and CellQuest Pro software (Becton Dickinson Biosciences (BDB), San Diego, CA). The EGFP expression was also visualised using a fluorescence microscope (IX71; Olympus, Tokyo, Japan) at 2 months after transduction.

### RNA isolation and analysis

Total RNA was isolated using TRIzol reagent (Ambion; Thermo Fisher Scientific). In HeLa IVS2-654 and HeLa EGFP-654 cells, pre-mRNAs splicing were analysed by reverse transcription polymerase chain reaction (RT-PCR) using rTth DNA polymerase (Applied Biosystems, Thermo Fisher Scientific) with Cy5-deoxycytidine triphosphate (Cy5-dCTP) (GE Healthcare, Amersham, UK). For β-globin mRNA analysis (Fig. S1A), forward (primer c; 5′-GGA CCC AGA GGT TCT TTG AGT CC-3′) and reverse (primer d; 5′-GCA CAC AGA CCA GCA CGT TGC CC-3′) primers were used. For EGFP mRNA analysis forward (Fig. S1B), (primer e; 5′-CGT AAA CGG CCA CAA GTT CAG CG-3′) and reverse (primer f; 5′-GTG GTG CAG ATG AAC TTC AGG GTC-3′) primers were used. The PCR was performed for 18 cycles.

In CD34 + cells, splicing of β^IVS2-654^-globin pre-mRNA was determined by RT-PCR. Reverse transcription was performed with oligo(dT) and RevertAid First Strand cDNA synthesis (Thermo Scientific; Thermo Fisher Scientific) followed by 18 cycles of PCR step containing Cy5-dCTP using β^A^-globin specific forward primer (primer a; 5′-GAA CGT GGA TGA AGT TGG TGG TG-3′) and reverse primer (primer b; 5′-CAC AGA CCA GCA CGT TGC CC-3′). The RT-PCR products were separated on 10% non-denaturing polyacrylamide gels and visualised using a Typhoon 9400 Imager (GE Healthcare). The ImageQuant TL analysis software (GE Healthcare) was used for quantification analysis.

### Vector copy number analysis

Genomic DNA was extracted using TRIzol reagent (Ambion). Vector copy number of U7.BP + 623 snRNA lentiviral vector was assessed by quantitative real-time PCR (qPCR) using primers specific for U7.BP + 623 snRNA gene (forward primer located on BP + 623 SSO sequence; 5′- GAG GCC TTT CCG CAT GTT A-3′ and reverse primer located on U7; 5′-CGT AGA ATT CAG GGG TTT TCC GAC CGA-3′) and human β-actin gene (forward primer; 5′-AGA CCT GTA CGC CAA CAC AG-3′ and reverse primer 5′-CCA GGG CAG TGA TCT CCT TC-3′). Real-time PCR was performed using SYBR® Green PCR Master Mix (Applied Biosystems).

### Flow cytometric analysis of erythroid cell differentiation

Erythroblast differentiation was analysed by FACSCalibur flow cytometer and CellQuest Pro software. The cells were stained with antibodies directed against two erythroblast markers, allophycocyanin (APC)-conjugated anti-human CD235a (BDB) and phycoerythrin (PE)-conjugated anti-human CD71 (BioLagend, San Diego, CA). The erythroblast stages were characterised by the expression patterns of CD235a and CD71^19^.

### Haemoglobin analysis

Haemoglobin composition of erythroid progenitor cells was analysed using an automatic HPLC system, Variant II (Bio-Rad)

### Statistical analysis

Correlation analysis between the percentage of β^A^-globin mRNA correction and haemoglobin A levels of erythroid progenitor cells after U7.BP + 623 treatment was performed using the Spearman rank method. Erythroid differentiation of erythroid progenitor cells between U7.BP + 623 versus SCR-treated groups were analysed using a paired student’s t-test. A *p*-value of less than 0.05 was considered to be statistically different.

## Supplementary information

Supplement information
