# Supplementary material for: Restoration of correct βIVS2-654-globin mRNA splicing and HbA production by engineered U7 snRNA in β-thalassaemia/HbE erythroid cells
Source: Sci Rep. 2019 May 21;9:7672. doi: 10.1038/s41598-019-43964-3 (PMC6529457; doi:10.1038/s41598-019-43964-3)
Supplement: Supplementary file 1 — Supplement information [file 41598_2019_43964_MOESM1_ESM.pdf]

Restoration of correct $\beta^{IVSII-654}$-globin mRNA splicing and HbA production by engineered U7 snRNA in β-thalassaemia/HbE erythroid cells.

Tiwaporn Nualkaew[1,+], Natee Jearawiriyapaisarn[1,+], Suradej Hongeng[2], Suthat Fucharoen[1], Ryszard Kole[3], Saovaros Svasti[1, 4, *]

[1]Thalassemia Research Center, Institute of Molecular Biosciences, Mahidol University, Nakhon Pathom, Thailand

[2]Departments of Pediatrics, Faculty of Medicine Ramathibodi Hospital, Mahidol University, Bangkok, Thailand

[3]Ercole Consulting, Chapel Hill, NC 27514, USA

[4]Department of Biochemistry, Faculty of Science, Mahidol University, Bangkok, Thailand

[+] These authors contributed equally to this study.

[*] **Corresponding author:**

Saovaros Svasti, Ph.D.

Thalassemia Research Center, Institute of Molecular Biosciences, Mahidol University, 25/25 Phuttamonthon 4 Road, Salaya, Nakhon Pathom 73170, Thailand. Tel: +66-2889-2557, Fax: +66-2889-2559,

E-mail: saovaros.sva@mahidol.ac.th, stssv@yahoo.com

Word count: abstract 196 words, methods 1,054 words, main text 2,544 words

Number of figure: 4

Number of supplement figure: 2

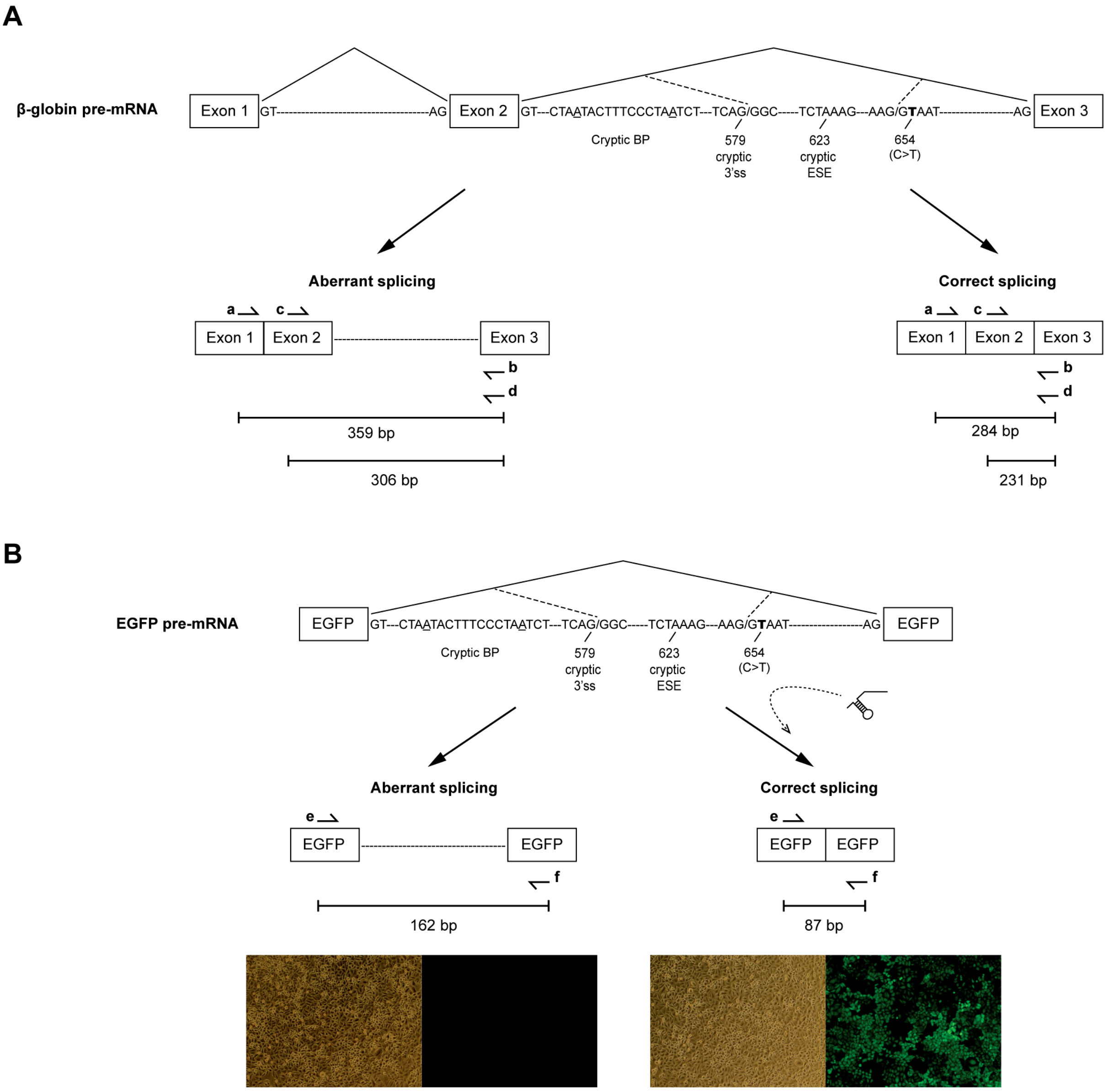

Figure S1. Splicing of human β$^{IVS2-654}$-globin pre-mRNA and EGFP-IVS2-654 pre-mRNA. (A) Splicing pathway of human β$^{IVS2-654}$-globin pre-mRNA. Aberrant β-globin pre-mRNA splicing resulted in retention a part of intron 2 into mature β-globin mRNA (left). Correct β-globin pre-mRNA splicing resulted in functional protein of β-globin chain (right). The sequence of splicing elements in intron 2 are shown. (B) Splicing pathway of EGFP pre-mRNA splicing in HeLa EGFP-654 cells. Interruption of EGFP coding sequence by the IVS2-654 β-globin intron 2 prevents EGFP expression. Correction of aberrant EGFP pre-mRNA splicing by engineered U7 snRNA restores EGFP expression provide a positive readout for antisense activity. Solid lines represent correct splicing and dashed lines represent aberrant splicing. Primers, half-headed arrows.

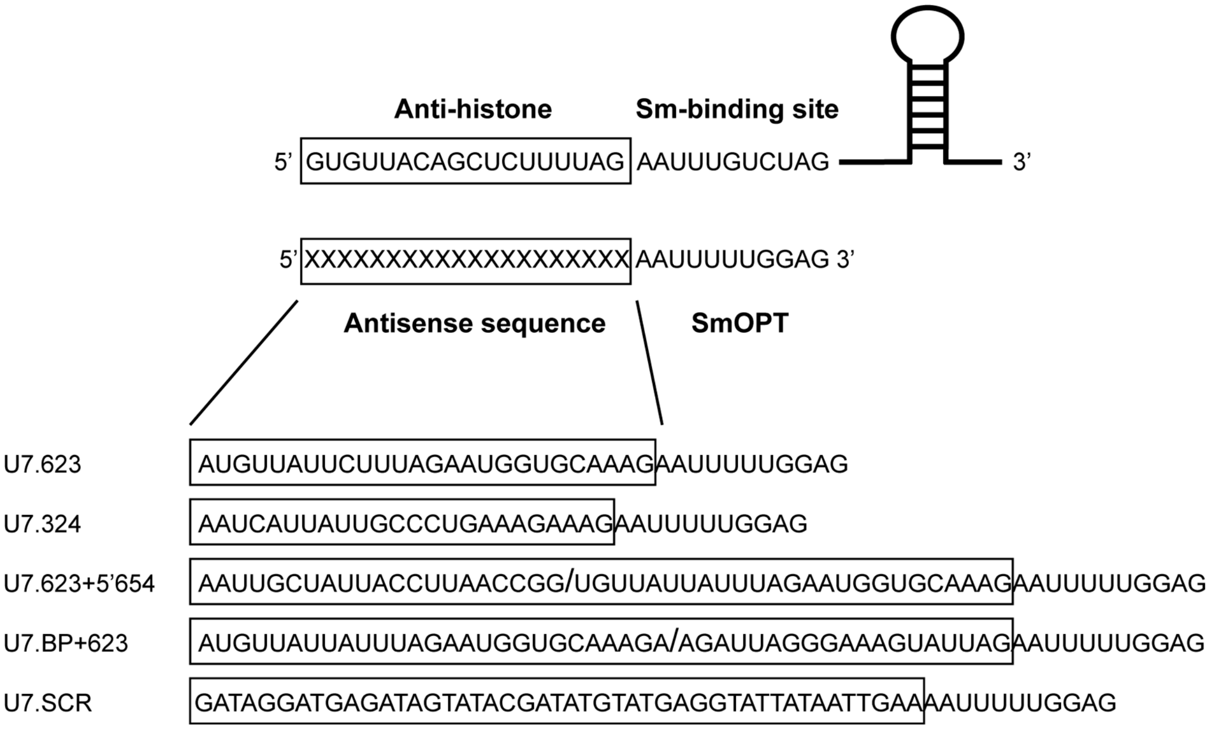

Figure S2: Structure of Modified antisense U7 snRNA. The anti-histone sequence, first 18 nucleotides, of 62-nt long U7 snRNA is replaced with antisense sequence and the Sm-binding site (AAUUUGUCUAG) is replaced with the consensus Sm-binding sequence derived from the major spliceosomal snRNPs (SmOPT; AAUUUUUGGAG). Antisense sequences are indicated in boxes.
